# Level of completion of the maternal health care continuum and its determinants among rural women in East Shoa Zone, Eastern Ethiopia: A multilevel analysis

**DOI:** 10.1371/journal.pone.0356392

**Published:** 2026-08-18

**Authors:** Serbesa Dereje Degaga, Solomon Shiferaw, Girma Taye Aweke

**Affiliations:** School of Public Health, College of Health Sciences, Addis Ababa University, Addis Ababa, Ethiopia; Wollega University, ETHIOPIA

## Abstract

**Background:**

The maternal continuum of care (CoC) is a key strategy for reducing maternal and neonatal morbidity and mortality. In this study, CoC completion was defined as receiving at least four antenatal care visits, skilled birth attendance and postnatal care within 48 hours after chilbirth. Despite continued national efforts to improve maternal health service utilization, CoC completion remains low in Ethiopia, particularly in rural areas. This study assessed the level of CoC completion and its associated factors among rural women in East Shoa Zone, Eastern Ethiopia.

**Methods:**

A community-based cross-sectional study linked to health facility data was conducted from November 5 to December 5, 2024. Using multistage sampling, 1,038 women who had given birth within the 12 months preceding the interview date were selected. Household data were collected through face-to-face interviews and facility readiness was assessed by direct observation using a SARA-adapted checklist. Multilevel logistic regression was used to identify factors associated with CoC completion. Results were reported as adjusted odds ratios with 95% confidence intervals.

**Results:**

Among 1,038 women invited to participate, 997 completed the interview, yielding a 96.1% response rate. Overall, 23.2% (95% CI: 20.6, 25.9) of women completed the maternal continuum of care. CoC completion was associated with secondary or higher education (AOR = 3.27; 95% CI: 1.37,7.79), early ANC initiation (AOR = 5.16; 95% CI: 3.01,8.83), knowledge of pregnancy danger signs (AOR = 3.89; 95% CI: 1.81,8.37), high facility readiness (AOR = 5.19; 95% CI: 1.48,18.16), travel time of less than one hour (AOR = 1.87; 95% CI: 1.01,3.17), and higher household wealth (AOR = 2.57; 95% CI: 1.41,4.67).

**Conclusion:**

Completion of the maternal continuum of care among rural women in East Shoa Zone was low with substantial dropout across successive stages. Both individual and health-system-level factors influenced CoC completion. Context-specific multilevel interventions should combine demand-side strategies that improve women’s awareness, preparedness, decision-making capacity and early initiation of maternal health care with supply-side measures that strengthen health facility readiness.

## Introduction

Globally, maternal mortality remains a major public health concern. Although motherhood is generally a source of joy for families particularly for women, each pregnancy in low-income countries presents significant risks to both women and newborns [1]. Approximately one woman dies every two minutes from pregnancy and childbirth related causes. The burden remains disproportionately concentrated in low and middle-income countries where about 92% of maternal deaths occurred. Sub-Saharan Africa alone accounted for approximately 70% of global maternal deaths. Ethiopia also continues to experience a substantial maternal mortality burden with an estimated maternal mortality ratio of 195 deaths per 100,000 live births in 2023. Although this reflects progress over time, the level remains markedly above the Sustainable Development Goal target of fewer than 70 maternal deaths per 100,000 live births by 2030 [2]. Global strategies recommend continuum of maternal health care as one of the key strategies for reducing maternal and newborn morbidity and mortality in LMICs [3]. Eighty percent (80%) of maternal deaths worldwide could be prevented with the provision of timely and quality healthcare during pregnancy, childbirth, and the postnatal period [4]. Achieving approximately 99% coverage of the complete maternal healthcare continuum also could avert an estimated 41%–72% of neonatal deaths. [5].

To ensure maternal continuum of care, Ethiopia has implemented several national strategies such as the National Reproductive, Maternal, Newborn, Child, and Adolescent Health (RMNCAH) Strategy (2016–2020) and the Health Sector Transformation Plan II (HSTP II, 2020–2025). These frameworks emphasize improving access to and utilization of the maternal health continuum of care, particularly antenatal care (ANC), skilled birth attendance, and postnatal care.

In addition, numerous programmatic strategies have been introduced to promote continuity across the maternal care pathway. These include household-based identification and registration of pregnant women by Health Extension Workers, pregnancy conferences to support coordinated follow-up and birth preparedness, strengthened referral linkages between health posts and health centers and the provision of key maternal health services such as the first and fourth antenatal care contacts and skilled delivery care at facilities beyond basic health posts to enhance the quality of maternal health care.[6–8].

Despite these interventions, ensuring a consistent provision of the maternal COC remains a significant challenge in Ethiopia. Studies reveal that out of those women who initiate ANC, merely 6.6% complete the COC, 29.96% women received ANC 4th dropped from institutional delivery and 81.78% who delivered at facility dropped from postnatal care in Ethiopia [9].

Failure to complete the maternal health continuum of care can lead to several adverse outcomes for both mothers and infants including increased maternal and neonatal morbidity and mortality, higher rates of obstetric complications, and overall poorer health outcomes. This is often due to untreated conditions such as anemia, infections, and hypertensive disorders which can have severe repercussions during pregnancy, childbirth, and the postpartum period. Mothers who do not receive the complete continuum of maternal care are at greater risk of complications during pregnancy and childbirth. In turn, these complications may contribute to increased maternal and neonatal mortality [10].

Previous Ethiopian studies using national and regional data have identified factors such as maternal education, household wealth, parity, pregnancy intention, early ANC initiation, and geographic accessibility as key determinants of completing the maternal continuum of care [9,11]. Multilevel analyses also highlight the influence of contextual and service delivery factors [12,13]. However, most evidence relies on secondary survey data often based on women’s five-year retrospective recall. Limited attention has also been given to objectively measuring health facility readiness, particularly in rural settings This gap may constrain understanding of contextual health system factors essential for designing effective maternal health strategies. Therefore, this study aimed to assess the level of completion of the maternal continuum of care (CoC) and factors associated with its adherence by linking household and health facility data for a comprehensive understanding.

## Methods and material

### Study design and setting

A community-based cross-sectional study was conducted from November 5 to December 5, 2024 to examine determinants of the completion of continuum of maternal healthcare services among rural women in East Shoa Zone, Eastern Ethiopia. The zone is located approximately 100 km southeast of Addis Ababa covering an area of 10,232 km^2^ within the Great Rift Valley. It has an estimated population of 1,386,490 of whom 306,830 are women of reproductive age. Administratively, it comprises 11 woredas, 2 town administrations, and 292 kebeles [14]. The study setting is characterized by dispersed rural settlements and travel-time barriers to health facilities which may affect women’s continuity of maternal healthcare utilization [15]. Health services are delivered within Ethiopia’s three-tier healthcare delivery system with potential variability in referral linkages and facility readiness across service levels [16]. The public health service infrastructure comprises 3 primary hospitals, 51 health centers, 5 comprehensive health posts, and 227 basic health posts [14]. Despite this service-delivery structure, maternal health service coverage remains substantially lower than the corresponding regional averages. According to the DHIS2 routine health service report, antenatal care, skilled birth attendance, and early postnatal care coverage were 71%, 52%, and 66%, respectively, compared with the regional averages of 97%, 70%, and 94% [17]..

### Study population and eligibility criteria

The source population consisted of women residing in rural areas of East Shoa Zone, Oromia Region who had given birth during the last 12 months preceding the survey period. The study population included all eligible women in the selected kebeles who had given birth during the 12 months before the interview date. Women who were critically ill or had lived in the kebele for less than 12 months at the time of data collection were excluded.

### Sample size calculation and sampling procedure

The required sample size was calculated using a single-population proportion formula, assuming an estimated prevalence of 21.5% for completion of the maternal continuum of care [18], and a 95% confidence level. A 4% margin of error (d = 0.04) was used instead of 5% to increase the precision of the estimate, acknowledging the associated increase in sample size.

To account for clustering, a design effect was incorporated into the sample-size calculation. Although the design effect is commonly estimated as DEFF = 1 + (m − 1) ρ, no reliable ICC estimate was available from a closely comparable study. Assigning an arbitrary value to ρ could have resulted in an inaccurate adjustment. Therefore, a conservative design effect of 2.0 was adopted to accommodate the anticipated variation in kebele sizes and the context-specific nature of the ICC. Finally, we included a 10% allowance for non-response. This yielded a final calculated sample size of 901 participants. Following establishment of the sampling frame and proportional allocation across kebeles, eligible women selected through systematic random sampling were traced to their households for participation.

During field implementation, 1,038 eligible women were identified and recruited from the sampling frame, of whom 997 completed the interviews. During the house-to-house survey, public health facilities providing at least basic maternal health services to the selected communities were also identified. Health centers that had been operational for at least one year prior to the data collection period were eligible for inclusion in the facility-level assessment. A service utilization–based linkage approach was used to integrate household and health facility data. Women were asked to identify the primary public health facility where they received maternal health services during their most recent pregnancy. Based on these reports, 12 public health facilities specifically health centers were included in the facility survey. Each woman was linked to the readiness score of the reported facility. This reflects the predominantly rural PHCU-based service delivery context where a catchment health center serves as the principal point of contact and referral hub for maternal health services.

A multi-stage sampling technique was employed to select study participants. In the first stage, four rural districts were randomly selected from the total of eleven rural districts in East Shoa Zone. In the second stage, 34 kebeles (hereafter referred to as clusters) were selected using simple random sampling. Kebele is the lowest administrative unit in Ethiopia. It represents a defined geographic community served by a local health post. In this study, kebeles were used as clusters in the multi-stage sampling procedure. It also reflects the local service and social context in which women reside and receive maternal health services.

In the third stage, within each selected kebele, a complete listing of eligible women who gave birth in the 12 months preceding the survey date was conducted in each selected kebele. The listing was prepared using family folders and registration logbooks maintained at health posts. In the Ethiopian Health Extension Program, family folders are household-level records maintained by Health Extension Workers at health posts. These records contain basic information on household members, including demographic characteristics, reproductive history, maternal and child health status, and service utilization. Together with health post registration logbooks, they were used to identify eligible women and construct the sampling frame. This process identified a total of 2,076 eligible women across 34 kebeles, which constituted the sampling frame. The number of eligible women varied across kebeles, ranging from 32 to 124, with an average of approximately 60 women per kebele. This variation reflects differences in population size across clusters and was accounted for through proportional allocation during sampling.

The required sample size (n = 901) was proportionally allocated to each kebele based on the number of eligible women identified. Within each kebele, a systematic random sampling technique was applied. The sampling interval was determined by dividing the total number of eligible women by the allocated sample size, yielding an interval of approximately k ≈ 2.3. For practical implementation, a fixed interval of k = 2 was used, and every second eligible woman was selected following a randomly chosen starting point. This procedure resulted in the selection of 1,038 women across all kebeles. Of these, 997 women completed the interviews, yielding a response rate of 96.1%.

### Data collection and procedures

At the household level, data were collected through face-to-face interviews using structured questionnaires administered during home visits. Health facility data were obtained through direct observation using standardized checklists. The health facility assessment checklist was adapted from the World Health Organization’s Service Availability and Readiness Assessment (SARA) tool. It covered six key domains: general amenities, basic equipment, standard precautions for infection prevention, diagnostic capacity, essential medicines and commodities, and staff and guidelines.

Data collection was conducted electronically using the KoboToolbox platform to enhance accuracy and efficiency. The questionnaires and checklists were developed based on a comprehensive review of relevant literature, national maternal and child health (MCH) guidelines, World Health Organization (WHO) recommendations, and the Ethiopian Demographic and Health Survey (EDHS) [19,20]. These tools were translated into the local languages, Afan Oromo and Amharic, and pre-tested in a similar setting to ensure clarity and contextual relevance. Necessary adaptations were made based on the pre-test findings.

Twenty trained data collectors with prior experience in survey implementation and four supervisors with a Master’s degree in Public Health (MPH) were recruited. All field personnel received a three-day intensive training on the study objectives, ethical considerations, and data collection procedures.

### Measurement

The maternity continuum of care (CoC) was defined as complete when a woman received all essential components of maternal health services, including a minimum of four antenatal care (ANC) visits, skilled birth attendance by a trained health professional such as a physician, health officer, nurse, or midwife, and postnatal care (PNC) provided within the first 48 hours following delivery [21]. The antenatal care component of the continuum of care was operationalized using a threshold of four or more ANC visits (ANC4+). Although the World Health Organization’s 2016 guideline recommends a minimum of eight ANC contacts, ANC4 + was retained in this study to ensure consistency with Ethiopia’s routine monitoring system, including DHIS2 and national RMNCAH indicators. It also facilitated comparison with previous studies conducted in similar settings.However,the ANC4 + category includes all visits beyond four, and therefore also captures women who attended eight or more ANC contacts. For analytical purposes, completion of the continuum of care was treated as a binary variable and coded as “1” if all three components were fulfilled and “0” otherwise.

Knowledge of pregnancy danger signs was assessed by asking participants to name known danger signs. Women who identified at least two of the four key danger signs (vaginal bleeding, severe headache, blurred vision, and swelling of the face or legs) were categorized as knowledgeable. Those who identified fewer than two were considered not knowledgeable [22].

Adequacy of ANC contents was assessed based on women’s self-reported receipt of ten essential components recommended by the World Health Organization (WHO) [23], as collected through a household survey. The components included measurement of body weight and blood pressure, urine and blood testing, administration of tetanus toxoid vaccination and deworming medication, provision of iron and folic acid supplements, and counseling on birth preparedness and recognition of key obstetric danger signs. The contents was classified as adequate if a woman reported receiving at least eight out of the ten specified ANC service components at least once during her most recent pregnancy. Women who received fewer than eight components were classified as having inadequate ANC contents [24,25].

Explanatory variables were selected based on established conceptual frameworks and prior empirical evidence on determinants of maternal continuum of care utilization. The variables were classified as individual, household, and contextual-level factors. Individual-level variables included maternal age, educational status, parity, pregnancy intention, timing of antenatal care initiation, adequacy of antenatal care content, knowledge of pregnancy danger signs, and travel time to the nearest health facility. Household wealth status was measured at the household level but assigned to individual women and analyzed as a Level 1 explanatory variable. The contextual-level variable was health facility readiness which was linked to each woman’s reported primary source of maternal health care and represented the service-delivery environment. Variables were structured across two analytical components within a multilevel framework. Level-1 variables captured both individual and household characteristics, while Level-2 components captured clustering at the kebele level and broader contextual influences.

The multilevel structure of the analysis was defined by clustering of women within kebeles, with random intercepts specified at the kebele level to account for between-kebele variation and unobserved contextual heterogeneity. In addition, health facility readiness was included as a contextual service-delivery factor linked to each woman’s reported source of maternal health care, reflecting differences in the service environment rather than a kebele-level administrative characteristic.

Facility readiness was measured using WHO SARA tools. Domain scores were calculated as the mean proportion of tracer items available within each domain. The domains included General amenities, Basic equipment, Standard precautions for infection prevention, Diagnostic capacity, medicines and commodities, and Staff and guidelines. The overall readiness index was then computed as the average of these domain scores, in line with WHO recommendations. Then readiness scores were linked to women based on the primary facility where they reported receiving most maternal services, reflecting the PHCU-based provision of maternal care in this predominantly rural setting.

Household wealth index was constructed using Principal Component Analysis (PCA) based on ownership of selected household assets and housing characteristics. Variables included household utilities, productive and non-productive assets, and housing conditions (e.g., source of drinking water, sanitation facilities, wall and flooring materials). The first principal component, which explained the largest proportion of variance among the asset variables, was retained to generate a composite household wealth score. The resulting scores were ranked and categorized into three groups: lower, medium, and higher wealth status. This tertile classification was used to enhance interpretability and to ensure adequate sample size within each category for multilevel analysis.

### Outcome variable

The outcome was completion of the continuum of maternal health care (CoC), defined as receipt of all three services: (i) ≥4 antenatal care visits (ANC4+), (ii) skilled birth attendance (SBA), and (iii) postnatal care (PNC) within 48 hours after delivery. To facilitate analysis, we also constructed stage-specific indicators and applied binary coding as follows:

Continued care at the pregnancy level. This measured whether a woman attained ANC4 + . It was coded 1 if she received at least four antenatal visits and 0 otherwise (n = 426). Continued care at the delivery level. This captured whether a woman attained both ANC4+ and SBA. It was coded 1 if she had ≥ 4 ANC visits and delivered with a skilled attendant, and 0 if she had ≥ 4 ANC visits but did not receive SBA (n = 315). Complete CoC at the postpartum level. This represented full completion of the continuum—ANC4 + , SBA, and PNC within 48 hours postpartum. It was coded 1 if all three components were met and 0 otherwise (n = 217).

### Data processing and analysis

Data were processed and analyzed using STATA version 17. Descriptive analysis was performed to summarize the utilization patterns of key maternal health services across the continuum of care. Retention and drop-out rates between successive service components were calculated to describe women’s progression along the continuum of care (ANC, skilled birth attendance, and postnatal care) and to identify stage-specific gaps in service utilization. Sampling weights were calculated as the inverse of the probability of selection at each stage: woreda, kebele, and household. Kebele selection probabilities were determined using probability proportional to size. Weights were normalized to preserve the effective sample size and applied to descriptive analyses using Stata’s survey (svy) commands which account for clustering at the kebele level.

Although the maternal continuum of care can be conceptualized as a sequential pathway across pregnancy, childbirth, and the postpartum period, the primary analytical outcome in this study was full completion of the continuum of care. Therefore, binary multilevel logistic regression was used to identify explanatory variables associated with complete CoC. Stage-specific descriptive indicators and regression models were also examined as secondary analyses to support interpretation of continuation from ANC4+ to skilled birth attendance and early postnatal care.

To account for the hierarchical structure of the data where women nested within kebeles, a two-level random-intercept logistic regression model was fitted. Women were considered Level 1 units, while kebeles were considered Level 2 units. Individual-level variables included maternal age, education, parity, pregnancy intention, timing of ANC initiation, adequate ANC contents, knowledge of danger signs, and travel time to the nearest health facility. Household-level characteristics, including household wealth status were measured at the household level but assigned to individual women and analyzed as Level 1 explanatory variables.

This approach was used because the woman was the primary unit of analysis, and households were not modeled as a separate random-effect level. The random intercept at the kebele level was included to account for intra-kebele correlation and unobserved contextual heterogeneity. This method adjusts for intra-cluster correlation and enables partitioning of variance into within- and between-cluster components. [26]. Three sequential two-level random-intercept logistic regression models were fitted to examine factors associated with completion of the continuum of care.

Model 0 (Null model) included no predictors and was used to quantify between-community variance:


$$\begin{array}{c} \text{Logit }(\text{Pij})\;=\beta \text{0}\;+\text{ u0j },\text{ with u0j }\sim \text{ N}(\text{0},\;\sigma^{\text{2}}\_\text{u}) \end{array}$$

Model 1 (Individual level) added individual-level explanatory variables I_ij_.


$$\begin{array}{c} \text{Logit }(\text{Pij})\;=\;\beta_{\text{0}}\;+\;\beta_{\text{1}}{\text{I}}_{\text{ij}}\;+\;{\text{u}}_{\text{0j}} \end{array}$$

Model 2 (Individual + contextual factor) further included contextual service-delivery factor, i.e., health facility readiness linked to each woman’s reported source of care. C_j_.


$$\begin{array}{c} \text{Logit }(\text{Pij})\;=\;\beta_{\text{0}}\;+\;\beta_{\text{1}}{\text{I}}_{\text{ij}}\;+\;\beta_{\text{2}}{\text{c}}_{\text{j}}\;+\;u_{\text{0j}} \end{array}$$

Where, P_ij_ is the probability of the outcome of interest for woman i in the community j; β_0_is the fixed intercept (overall mean on the log-odds scale); β_1_ and β_2_ are fixed-effect coefficients for the individual and community-level predictors respectively; I and C refer to individual and community-level independent variables respectively, and u_0j_ is the community-specific random intercept capturing unobserved between-community heterogeneity. Fixed effects were expressed as odds ratios (ORs) with 95% confidence intervals (CIs) and p-values. Random effects were assessed using the intra-class correlation coefficient (ICC) [27]. The ICC was calculated as:


$$\begin{array}{c} \text{ICC}=\;{\sigma \text{1}}^{\text{2}}\;/\;({\sigma \text{1}}^{\text{2}}+\;\pi^{\text{2}}/\text{3}) \end{array}$$

In this expression, σ1^2^ denotes the between-community (level-2) variance, π represents the mathematical constant pi ≈ 3.14159 and π^2^/3 denotes the assumed individual-level (level-1) residual variance. In logistic multilevel models with binary outcomes, the level-1 residual variance is not estimated directly; instead, it is assumed to follow a logistic distribution with fixed variance π^2^/3 ≈ 3.29. [26].

### Ethical considerations

Ethical approval for this study was obtained from the Institutional Review Board of the College of Health Sciences, Addis Ababa University (Protocol No. 059/24/SPH, dated 01 November 2024). Written informed consent was secured from all participants prior to their participation in the study. To ensure privacy and confidentiality, personal identifiers were intentionally omitted from the data collection form.

## Results

### Socio-demographic characteristics of participants

A total of 1,038 women were invited to participate of whom 997 completed the interviews, resulting in a response rate of 96.1%.The majority of the respondents 651 (65.3%) were between 25 and 34 years old, with a mean age of 26.58 years (SD ± 4.319). Most participants were married 984 (98.7%) and predominantly identified as Oromo in ethnicity 863 (86.6%).

Regarding educational attainment, nearly half of the women 450 (45.14%) had no formal education. In terms of employment, a substantial proportion of their husbands 808 (81.04%) were engaged in farming. With respect to household wealth distribution, 420 women (42.13%) belonged to the lower wealth quintile. Table 1

**Table 1:** Socio-demographic characteristics of women who gave births within the past 12 months in East Shoa Zone, Eastern Ethiopia, 2024 (n = 997).

Variable	Categories	Frequency (n)	Percent (%)
Age of women (years)	15-24	272	27.28
	25-34	651	65.3
	35-49	74	7.42
Marital status	Married	984	98.7
	Others	13	1.3
Mothers educational status	Cannot read and write	210	21.06
	Can read and write	240	24.07
	Primary education	409	41.02
	Secondary and above	138	13.84
Mothers’ occupation	Farmer	712	71.41
	Daily worker	100	10.03
	Merchant	61	6.12
	Employed	31	3.11
	House wife	93	9.33
Education status of the husband	Cannot read and write	232	23.27
	Can read and write	313	31.39
	Primary education	360	36.11
	Secondary and above	92	9.23
Husbands’ Occupation	Farmer	808	81.04
	Daily worker	84	8.43
	Merchant	31	3.11
	Employed	51	5.12
	Others	23	2.31
Religion	Orthodox	491	49.25
	Catholic	21	2.11
	Protestant	226	22.67
	Muslim	220	22.07
	Wakefata	39	3.91
Ethnicity	Oromo	863	86.56
	Amhara	97	9.73
	Others	27	3.71
Family wealth Index	Lower	420	42.13
	Medium	183	18.36
	Higher	394	39.52

### Obstetrics and reproductive health characteristics

Approximately three-fifths of the women demonstrated adequate knowledge of obstetric and reproductive health services. Approximately half of the participants, 507 (50.9%), were multiparous.. A substantial proportion of the pregnancies, 728 (73.02%), were reported as both planned and intended. Furthermore, 518 women (51.96%) had a birth-to-pregnancy interval of two years or less. Obstetric complications were reported by 115 women (11.53%), while 138 (13.84%) had experienced adverse fetal outcomes in previous pregnancies. Table 2

**Table 2:** Obstetric and reproductive health characteristics of women who delivered within the past 12 months in East Shoa Zone, Eastern Ethiopia, 2024.

Variable	Categories	Frequency (n)	Percent (%)
Parity	Primipara (1)	378	37.91
	Multipara (2 –4)	507	50.85
	Grand multipara (5 or more)	112	11.23
Birth interval	Less than 24 months	518	51.96
	25-36 months	275	27.58
	37-60 months	198	19.86
	more than 60 months	6	0.6
Planned pregnancy	Yes	648	64.99
	No	349	35.01
Had obestetric complications	Yes	115	11.53
	No	882	88.47
History of adverse pregnancy outcomes	Yes	138	13.84
	No	859	86.16
Previous use of family planning	Yes	708	71.01
	No	289	28.99
Knowledge of at least 2 danger signs	Yes	589	59.08
	No	408	40.92
Mode of delivery	SVD	841	84.35
	CS/Assisted	156	15.65

### Descriptive presentation of maternal health care services utilization

Among all participants, 736 women (73.82%) received at least one antenatal care (ANC) visit during their most recent pregnancy, while 426 (45.3%) attained minimum of four or more ANC contacts. Of those who initiated ANC, only 165 (22.4%) began care within the first trimester. Furthermore, among women who received at least one ANC visit, 172 (23.4%) did not receive adequate ANC service components recommended by the World Health Organization.

With regard to delivery services, only half of the most recent 515(51.65%) births were attended by skilled birth attendants. The predominant reasons for home delivery included the sudden onset of Labor 208(43.70%), lack of transportation 159(33.4%), and poor quality of service 68(14.3%). Additionally, 115 women (11.53%) experienced complications during or immediately after childbirth. The most commonly reported complications were severe vaginal bleeding 40 (34.8%), abdominal pain 39 (33.9%), severe headache 11 (9.6%), fever 11 (9.6%) and blurred vision 5 (4.4%). Regarding Postnatal care only half (50.65%) of the women reported receiving at least one postnatal care (PNC) service. Nevertheless, only 3.13% of women attended postnatal care two or more times. Table 3

**Table 3:** Distribution of study participants by their maternal health care utilization in East Shoa Zone, Eastern Ethiopia, 2024.

Variable	Categories	Frequency (n)	Percent (%)
Received any ANC	Yes	736	73.82
	No	261	26.18
Had 4 and more ANC visits	Yes	426	42.73
	No	571	57.27
Time of booking for ANC	Within 12 weeks	129	17.53
	After 12 weeks	607	82.47
Content of ANC	Adequate	564	76.63
	Inadequate	172	23.37
Place of delivery	Health Facility	515	51.65
	Home	482	48.35
Received PNC	Yes	505	50.65
	No	492	49.35
Frequency of PNC	Once	489	96.8
	Two and more	16	3.2

### Completion of the maternal health care continuum

After applying sampling weights to account for the multistage design and clustering at the kebele level, 45.3% (95% CI: 42.1, 48.5) of women received ≥4 antenatal care visits, 33.0% (95% CI: 30.1, 36.0) received both ANC ≥ 4 and skilled birth attendance, and 23.2% (95% CI: 20.6, 25.9) completed the full continuum of maternal health care (ANC ≥ 4, SBA, and PNC within 48 hours). In absolute terms, 426 women received four or more antenatal care (ANC) visits. Among these, 315 subsequently delivered in a health facility with skilled birth attendance. However, only 217 women completed the full continuum of maternal health care, defined as receiving four or more ANC visits, skilled birth attendance, and postnatal care within 48 hours after childbirth.

**Fig 1:**
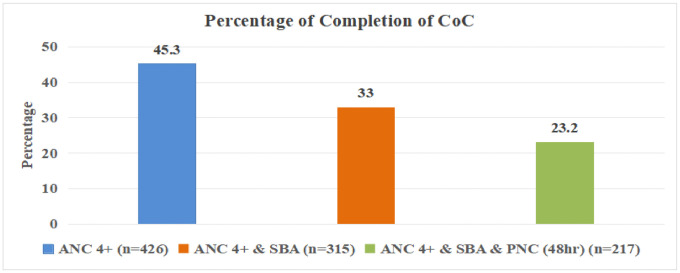
Proportion of women who completed the maternal continuum of care among those who gave birth in the 12 months preceding the survey in East Shoa Zone, Eastern Ethiopia, 2024.

The completion of the maternity continuum of care (CoC) varied significantly across age and parity categories. A chi-square test showed statistically significant differences in CoC completion across age groups (χ² = 14.06, p = 0.001). Women aged 25–34 years had the highest completion rate (25.2%), followed by those aged 15–24 years (16.5%), while the lowest rate was observed among women aged 35–49 years (10.8%). Similarly, CoC completion differed significantly across parity categories (χ² = 6.41, p = 0.04). Women with primiparity had a completion rate of 23.3%, compared with 22.7% among multiparous women, and 12.5% among grand multiparous women. Fig 1.

ANC = Antenatal Care (minimum four visits); SBA = Skilled Birth Attendance; PNC = Postnatal Care within 48 hours of delivery.

### Multilevel analysis

In the multilevel model analysis, we initially assessed whether the data warranted the inclusion of random effects at the kebele (cluster) level. The null model demonstrated substantial variation in the odds of completing the continuum of care across communities, supporting the application of a multilevel modeling approach. The observed mean cluster size was mˉ = 29 and the ICC was ρ = 0.435, implying DEFF = 1+ (mˉ − 1) ρ = 1+ (29 − 1)×0.435 = 13.18. The intraclass correlation coefficient (ICC) revealed that 43.5% of the total variance in the completion of the continuum of care was attributable to unmeasured differences between clusters. After adjusting for all study explanatory variables, the between-cluster variance decreased to 27.9% in Model 1 and further to 25.8% in Model 2. Nonetheless, the residual variance at the cluster level remained substantial. This suggests that the continued presence of unmeasured kebele-level influences on the completion of continuum of care services despite accounting for level-1 and level-2 explanatory variables. Table 4

**Table 4:** Random effects parameters for the three multilevel analysis models.

Random effect parameters	Null model	Model 1	Model 2
Variance (95% CI)	2.53(1.24, 5.18)	1.27(.49,3.29)	1.14(.49,3.29)
Rho-ICC	0.435	0.297	0.258
MOR	4.56	2.93	2.77
PCV	–	0.497	0.549
**Model fit statistics**			
Log-likelyhood (−2LL)	454.349	299.912	295.106

### Determinants of completion of the continuum of maternal health care

The adjusted multilevel model identified several significant determinants of completing the continuum of maternal health care. At the individual and household level (level-one), maternal education, timing of the first antenatal care (ANC) visit, history of poor fetal outcomes, and household wealth status were significantly associated with completion of the continuum of care. Women who attained at least secondary education were over 3 times more likely to complete the continuum of care compared to those with no formal education (AOR = 3.27; 95% CI: 1.37, 7.79).

Early initiation of ANC within the first 12 weeks of gestation was also significantly associated with completion of the continuum of care; women who booked ANC early had over five times higher odds of completing the continuum of care than those who initiated ANC later (AOR = 5.16; 95% CI: 3.01,8.83). Similarly, women with a history of poor fetal outcomes were 2.46 times more likely to complete the continuum of care (AOR = 2.46; 95% CI: 1.33, 4.51). Furthermore, those women in the higher wealth quintile had 2.57 times greater odds of utilizing all recommended maternal health services compared to women in the lower wealth quintile (AOR = 2.57; 95% CI: 1.41,4.67).

Proximity to a health facility was significantly associated with completion of the maternal continuum of care. Women living within one hour’s travel time to a health facility had higher odds of completing the continuum of care than those residing farther away (AOR = 1.87; 95% CI: 1.01, 3.17). In addition, women who received care from health facilities with higher readiness scores were substantially more likely to complete the continuum of care compared with those served by facilities with lower readiness levels (AOR = 5.19; 95% CI: 1.48,18.16).

Stage-specific regression models were also fitted to examine continuation across the maternal care pathway. Early ANC initiation was associated with ANC4 + , ANC4+ with skilled birth attendance, and complete continuum-of-care utilization, with the strongest association observed for complete CoC (AOR = 5.16; 95% CI: 3.01,8.83). Similar cross-stage associations were observed for history of poor fetal outcome, shorter distance to a health facility, and high facility readiness. Women’s autonomy and planned pregnancy were more strongly associated with later-stage outcomes. Women with autonomy had higher odds of ANC4+ with skilled birth attendance (AOR = 1.72; 95% CI: 1.15, 2.56) and complete CoC (AOR = 3.12; 95% CI: 1.89, 5.14). Women with planned pregnancies also had higher odds of ANC4+ with skilled birth attendance (AOR = 2.16; 95% CI: 1.40, 3.32) and complete CoC (AOR = 4.18; 95% CI: 2.17, 8.01). Table 5

**Table 5:** Primary and stage-specific multilevel logistic regression models for maternal continuum-of-care utilization among women who gave birth in the 12 months preceding the survey in East Shewa Zone, Ethiopia, 2024.

Variables	Category	AOR (95% CI)		
		Continued care at the pregnancy level (ANC4+)	Continued care at the delivery level (ANC4+ and SBA)	Complete CoC at the postpartum level (ANC4 + &SBA &PNC(48hr)
**Fixed Effects**				
**Individual and household level factors**				
Age of the mother	35 + years	1	1	1
	15-24	.60(.29, 1.25)	0.75(.34, 1.70)	1.19(.38, 3.73)
	25-34	1.05(.57, 1.98)	1.27(.63, 2.56)	1.72(.62, 4.74)
Women’s education status	Cannot read and write	1	1	1
	Can read and write	.95(.59, 1.54)	1.06(.62, 1.81)	1.29(.64, 2.65)
	Primary education	1.85(1.15, 2.97)*	2.17(1.32, 3.56)*	3.05(1.49, 6.26)*
	Secondary and above	1.52(.79, 2.93)	1.99(1.02, 3.91)*	3.27(1.37,7.79)*
Husband’s Occupation	Farmer	1	1	1
	Daily worker	1.18(.66, 2.12)	1.43(.79, 2.56)	0.99(.37,2.65)
	Merchant	.79(.32, 1.96)	.58(.23, 1.48)	0.43(.14,1.38)
	Employed	2.95(1.29, 6.74)*	2.64(1.17, 5.94)	2.58(1.05,6.33)*
	Other	1.40(.35, 5.60)	1.46(.33, 6.35)	1.20(.17, 8.28)
Family size	Five and more	1	1	1
	Less than five	1.13(.72, 1.78)	1.05(.64, 1.71)	1.44(.75,2.77)
Family wealth index	Low	1	1	1
	Medium	.73(.45, 1.19)	0.82(.49, 1.39)	1.14(.56,2.32)
	Higher	**1.82(1.18, 2.82)**	1.87(1.18, 2.93)	2.57(1.41,4.67)*
Women’s autonomy	No	1	1	1
	Yes	1.20(.83, 1.75)	1.72(1.15, 2.56)*	3.12(1.89,5.14)**
Planned pregnancy	No	1	1	1
	Yes	1.15(.78, 1.70)	2.16(1.40, 3.32)**	4.18(2.17,8.01)**
Age at first marriage	<18 years	1	1	1
	>= 18 years	1.25(.84, 1.86)	1.45(.96, 2.19)	2.69(1.51,4.80)*
waiting_time_to_see_medical staff	More than 1 hr	1	1	1
	Less than 30 min	.73(.35, 1.51)	.83(.39, 1.75)	1.40(.42,4.67)
	30 min-1 hr	.70(.35, 1.42)	.74(.36, 1.53)	0.65(.19,2.18)
Time of booking for ANC	After 12 weeks	1	1	1
	Within 12 weeks	2.62(1.70, 4.04)**	3.00(1.99, 4.56)**	5.16(3.01,8.83)**
Content of ANC	Inadequate	NA	NA	1
	Adequate			2.49(1.24,4.99)*
Knowledge of at least 2 danger signs	No	NA	NA	1
	Yes			2.82(1.71,4.63)**
Had obestetric complications	No	NA	NA	1
	Yes			0.59(.28,1.29)
Mode of transportation	On foot	1	1	1
	Ambulance	1.83(1.11, 3.02)*	1.62(.97, 2.74)	2.05(1.04,4.05)*
	Public transport	.87(.50, 1.53)	1.16(.65, 2.06)	1.43(.65,3.18)
	Cart	1.48(.53, 4.11)	2.99(1.08, 8.31)*	3.59(1.05,12.32)*
	Keraza	1.58(.74, 3.38)	1.35(.59, 3.09)	1.53(.50,4.69)
Mode of delivery	SVD	NA	NA	1
	CS/Assisted			2.99(1.65,5.46)**
Parity	Grand multipara (5 or more)	1	1	1
	Primipara (1)	1.26(.69, 2.27)	1.36(.71, 2.61)	1.53(.60,3.88)
	Multipara (2 –4)	1.23(.72, 2.11)	1.41(.77, 2.57)	2.12(.88,5.12)
Told about danger signs	No	NA	NA	1
	Yes			3.89(1.81,8.37)**
History of poor fetal outcome	No	1	1	1
	Yes	2.35(1.46, 3.78)**	2.49(1.54, 4.02)**	2.45(1.33,4.51)*
Distance to health facility	More than 1 hr	1	1	1
	Less than or equal to 1hr	1.82(1.28, 2.61)*	2.06(1.41, 3.01)**	1.87(1.01,3.17)*
**Contextual factor**				
Health facility readiness level	Low readiness	1	1	1
	Medium readiness	1.14(.84, 1.51)	1.13(.74, 1.40)	1.07(.29,3.88)
	High readiness	2.24(1.39, 3.87)*	1.71(1.26, 3.93)*	5.19(1.48,18.16)*
**Random effects**				
Variance (95% CI)		1.26 (**0.91, 1.77)**	1.07(0.52, 2.22)	1.14(.47,2.80)
Rho-ICC		0.327	0.246	0.258
Log-likelihood (−2LL)		521.083	480.748	295.106

* Statistically significant at <0.05, ** Statistically significant at P < 0.001, ICC:Intraclass correlation Coefficient;-2LL-Loglikelihood.

CI=confidence interval;1=reference.

## Discussion

This study assessed the level of completion of the maternal continuum of care and identified factors associated with completion among rural women in East Shoa Zone, Ethiopia. The findings showed that completion of the maternal continuum of care remained low. Overall, 23.2% of women completed the full continuum of maternal health care services. This indicates that nearly four out of five women did not receive the complete package of essential maternal health services.

The stage-specific findings also showed a progressive decline across the maternal care pathway. Although 45.3% of women received at least four antenatal care visits, only 33.0% received both ANC4+ and skilled birth attendance. The proportion further declined to 23.2% for complete continuum of care utilization. This decline indicates that women were lost from care at multiple transition points, particularly between antenatal care, skilled delivery, and timely postnatal care. The finding reflects substantial gaps in both service delivery and continuity of care. While antenatal care provides an important opportunity for counseling, risk identification, birth preparedness, and referral planning, its benefits may be limited if women are not retained through childbirth and the early postpartum period. Therefore, improving ANC attendance alone is unlikely to ensure completion of the maternal continuum of care. Programs should place greater emphasis on strengthening linkage, follow-up, and retention across all stages of maternal health service delivery.

The completion level observed in this study is comparable with findings from a multi-region Ethiopian study involving Amhara, Oromia, SNNP, and Tigray, and from West Shoa Zone of Oromia Region, where completion was reported as 21.5% [28,29]. However, the finding was higher than reports from Cambodia, 5% [28], Chad, 6.5% [29], Nigeria, 6.5% [30], Uganda, 10.7% [31], Gondar Zuria District, 6.9% [12], and Arba Minch Zuria Woreda, 9.7% [32]. It was also higher than completion rates reported in multilevel analyses of the 2016 Ethiopian Demographic and Health Survey, which documented completion rates of 5.17% and 6.56% in separate studies [9,33]. These differences may be related to variations in health service accessibility, quality of care, expansion of health facilities, availability of skilled health professionals, maternal health awareness, and programmatic emphasis on maternal health interventions.

However, the completion rate in this study was lower than findings reported from Sub-Saharan Africa, 35.81% [34], Ghana, 66% [33], and Egypt, 50.4% [35]. It was also lower than findings from Northwest Ethiopia, 47% [36], Southern Gondar, 37.6% [37], and East Gojjam, Ethiopia, 45% [3]. These disparities may be explained by variations in study setting, respondent education, geographic access, infrastructure, and health service availability. Some of the studies reporting higher completion rates were conducted in urban or town settings, while the present study included only rural women. Spatial analyses have also demonstrated clusters of low service utilization in regions such as Oromia, highlighting the influence of geographic inequities on the continuum of care [38].

Differences in how “completion of the continuum of care” is defined and measured may also explain variations across studies. In this study, completion required at least four ANC visits, skilled birth attendance, and postnatal care within 48 hours after delivery. However, some previous studies used at least one ANC visit and postnatal care within six weeks after birth to define completion. Therefore, women who had fewer than four ANC visits or received postnatal care after 48 hours were considered as dropouts in the present study. This stricter operational definition may have resulted in a lower estimated completion rate.

Maternal education was significantly associated with completion of the maternal continuum of care. Mothers with secondary education or higher were 3.27 times more likely to complete the maternity continuum of care compared with those with no formal education. It indicates that educated women may be better positioned to understand health information, recognize the value of completing all recommended services, and make informed decisions about continuing care. This finding aligns with studies conducted in Ethiopia, Pakistan, Zambia, and Nigeria [21,39–43]. The association may be explained by the role of education in improving health literacy, communication with health providers, autonomy, socioeconomic opportunity, and ability to overcome barriers to accessing services. These findings suggest that investment in girls’ and women’s education should be viewed as an important long-term strategy for improving maternal and child health outcomes.

Counseling on pregnancy danger signs was also associated with completion of the continuum of care. Women counseled on pregnancy danger signs were approximately 3.89 times more likely to complete the continuum than those who were not counseled. This implies that counseling is not only important for complication recognition, but also for keeping women connected to the health system throughout the maternal care pathway. It also indicates that counseling during ANC may create awareness, perceived need, and motivation for continued use of skilled delivery and early postnatal care. This observation is in agreement with evidence from Tanzania and Ethiopia [9,44–46]. This consistency may be partly explained by the role of danger-sign counseling in improving women’s awareness of potential complications, encouraging timely care-seeking, and supporting continued engagement with maternal health services. This finding underscores the importance of strengthening targeted ANC counseling as a practical intervention to improve early recognition of complications, timely use of care, and continuity across the maternal health care pathway.

Early initiation of antenatal care was one of the strongest predictors of complete continuum of care utilization. Mothers who initiated ANC within the first three months of pregnancy were 5.16 times more likely to complete the continuum of maternal health care than those who initiated care later. This reflects that early ANC serves as a critical entry point for sustained engagement with maternal health services. It indicates that women who begin ANC early have more opportunities to receive counseling, risk assessment, birth preparedness support, and linkage to delivery and postnatal services. In contrast, delayed pregnancy disclosure and late recognition of pregnancy may contribute to late ANC initiation and missed opportunities for early intervention [44]. This finding is supported by studies conducted in Sub-Saharan Africa, Kenya, and Ethiopia [28,47,48]. A plausible explanation is that early ANC may enable timely detection and management of complications, provision of tailored health education, and stronger linkage with the health system. The finding suggests that interventions that promote early pregnancy identification and early ANC booking among women, families, and communities are essential for improving completion of the maternal continuum of care.

Adequate ANC content was positively associated with completion of the maternal continuum of care. Women who received adequate ANC contents were 2.49 times more likely to complete the continuum of maternal health services. This highlights that the quality and substance of ANC contacts matter, not only the number of visits. ANC contacts that include counseling, risk assessment, birth preparedness, danger sign counseling, and linkage to delivery and postnatal services may help women remain engaged across the maternal care pathway.

This finding is similar to studies from Ethiopia showing that adequate ANC components and high quality ANC are associated with improved continuation to skilled birth attendance and other maternal health services [24,47]. Programmatically, this implies that improving the completeness of ANC content should be prioritized alongside increasing ANC attendance. However, this measure reflects selected ANC components and should be interpreted as an indicator of ANC content adequacy, not as a comprehensive measure of the full multidimensional quality of ANC.

Pregnancy intention was another important determinant of completion. Women with planned pregnancies were 4.18 times more likely to complete the full continuum of care than women with unplanned pregnancies. This shows that planned pregnancy may increase readiness to engage with maternal health services from early pregnancy through the postpartum period. It indicates that women with planned pregnancies may be more psychologically prepared, more likely to recognize pregnancy early, and better supported to seek care consistently. This finding is consistent with previous evidence showing the importance of planned pregnancy for maternal health service utilization [48,49]. The observed association may reflect greater family support, proactive health-seeking and better preparation for pregnancy and childbirth among women with planned pregnancies. Integrating family planning and reproductive counseling into maternal health programs may therefore improve continuity of care and maternal health outcomes.

Completion was also influenced by proximity to health facilities and facility readiness. Women living within a one-hour walk of a facility were 1.87 times more likely to complete the continuum than those requiring longer travel. This demonstrates that geographic access remains a major condition for sustained use of maternal health services in rural settings. It suggests that distance may affect not only initial service contact, but also women’s ability to continue care through delivery and the early postpartum period, when timely access is especially important. This finding aligns with evidence from Cambodia and Ethiopian studies emphasizing the importance of access and readiness for continuity of care [30,35,36]. Shorter travel time may reduce logistical, financial, and physical barriers to care, especially during labor and after childbirth. These findings highlight the need to improve equitable access, strengthen transportation systems, and ensure consistent care across all stages of the continuum.

Facility readiness emerged as a strong determinant of completion. Women receiving care from higher-readiness facilities were 5.19 times more likely to complete the continuum of care. This underscores that the service environment is central to whether women continue through the full maternal care pathway. It indicates that women may be more likely to complete care when facilities are able to provide reliable, comprehensive, and acceptable services. This finding concurs with evidence from Nepal, Tanzania, and Ethiopia [15,50,51]. The observed association may be related to the presence of essential infrastructure, skilled staff, reliable supplies, and better service organization in prepared facilities. These conditions may improve perceived quality, build trust, reduce missed opportunities, and encourage women to return for subsequent services. Health system planners and policymakers should therefore prioritize investments in infrastructure, supplies, staffing, and service readiness to improve completion of the maternal continuum of care and maternal and neonatal outcomes.

Although complete CoC was the main outcome of interest, stage-specific regression models were also examined to support interpretation of continuation across the sequential maternal care pathway. The results showed that early ANC initiation, history of poor fetal outcome, shorter distance to a health facility, and high facility readiness were associated with continued use of maternal health services across multiple stages of the continuum. This implies that completion of the maternal continuum of care is not influenced by a single factor at one service point, but by a combination of factors that support women’s retention across pregnancy, childbirth, and the early postpartum period. This pattern is consistent with recent studies from Ethiopia and other Sub-Saharan African settings, which reported that early ANC engagement, geographic access, service readiness, and shorter distance to a health facility influence continuation and dropout across the maternal care pathway [28,52].

Early ANC initiation may create opportunities for repeated counseling, risk identification, birth preparedness, and referral linkage. Similarly, previous poor fetal outcome may increase women’s perceived risk and motivation to continue care, while shorter distance and better facility readiness may reduce access barriers and improve confidence in the health system. Women’s autonomy and planned pregnancy were more strongly associated with later-stage continuation, including continuation from ANC4+ to skilled birth attendance and complete CoC. This suggests that decision-making power, pregnancy preparedness, and household support may be particularly important for moving from ANC attendance to skilled delivery and timely postnatal care [53].These findings highlight the need for interventions that promote early engagement with ANC while also strengthening retention mechanisms across the full maternal care pathway.

The relatively high ICC observed in this study indicates that a substantial proportion of variation in completion of the continuum of care was explained by differences between kebeles rather than individual characteristics alone. This suggests that community-level and health-system factors play an important role. Possible unmeasured contextual influences may include variation in health extension worker performance, referral functionality, local social norms, transport availability, and implementation strength of maternal health programs. These findings support the need for context-specific interventions at both community and health-system levels.

The findings have important implications for maternal health programming in rural settings. Interventions should not focus only on increasing ANC contact. They should also strengthen retention from ANC to skilled delivery and early postnatal care. Early pregnancy identification, appointment tracking, counseling on danger signs, birth preparedness, referral linkage, and postpartum follow-up should be strengthened. Health facilities should also be supported to maintain readiness through adequate supplies, equipment, essential medicines, trained providers, and functional referral systems.

Although ANC4 + was used in this study as an operational and programmatic threshold for measuring continuum-of-care completion, this should not be interpreted as suggesting that four ANC visits are sufficient for optimal antenatal care. Policy and programmatic recommendations should therefore be understood within Ethiopia’s routine monitoring context, while continuing to support the transition toward the WHO recommended minimum of eight ANC contacts. Maternal health programs should strengthen both the number and content of ANC contacts, while ensuring that women are retained across skilled birth attendance and timely postnatal care.

The findings also suggest the need for targeted support for women at higher risk of dropping out from the continuum. These include women with no formal education, women who initiate ANC late, women with unplanned pregnancies, women living far from health facilities, and women served by facilities with lower readiness. Community-based strategies involving health extension workers, women’s groups, and local community structures may help improve follow-up and retention across the maternal care pathway.

Future research should further examine why women discontinue care at different stages of the continuum. Qualitative studies may help explore social, cultural, economic, and health-system barriers to skilled delivery and early postnatal care. Longitudinal designs would be useful to better establish temporal relationships between explanatory variables and continuum-of-care completion.

### Study strengths and limitations

This study has several strengths. It used community-level data from rural women who gave birth within the 12 months preceding the survey. This reduced the potential for long recall periods compared with surveys using five-year birth histories. The study also linked household-level data with contextual factors, particularly health facility readiness, to comprehensively examine determinants of completion of the maternal continuum of care. In addition, the use of multilevel analysis accounted for clustering of women within kebeles and allowed assessment of between-community variation.

This study also has limitations. The use of self-reported maternal health service utilization may have introduced recall bias and social desirability bias. To reduce recall bias, the study included women who gave birth within the 12 months preceding the survey. The adequacy of ANC contents was assessed using selected service components, with all components weighted equally for analytical simplicity although their clinical importance may differ. Therefore, this measure should be interpreted as an indicator of ANC content adequacy rather than the full multidimensional quality of ANC care. The cross-sectional design also limits causal and temporal interpretation of the associations observed.

The empirical design effect of 13.18 exceeded the planning assumption of 2.0, indicating stronger within-community correlation than expected and a smaller effective sample size. Although this may reduce statistical power, the multilevel models accounted for the observed clustering. Future studies in similar settings should consider higher ICC assumptions or sensitivity analyses during sample-size planning. Linking women to a single facility based on their self-reported primary source of care may have introduced misclassification, particularly if services across pregnancy, delivery, and postnatal care were obtained from different facilities. However, the study was conducted in a rural PHCU-based context where maternal care is commonly coordinated through a dominant catchment facility. The retrospective design also did not allow verification of facility use at each contact, which may have led to non-differential misclassification of facility readiness.

The study used four or more antenatal care visits as the ANC component of continuum-of-care completion. Although ANC4 + was retained as an operational threshold because of its use in Ethiopia’s routine monitoring system, comparability with previous studies, and the potential difficulty of accurately recalling the exact number of contacts beyond four, it does not represent the current standard for optimal antenatal care. The WHO recommends a minimum of eight ANC contacts. The use of ANC4 + may therefore have overestimated continuum-of-care completion and underestimated the service gap that would have been observed under an ANC8 + definition. Accordingly, the reported prevalence should be interpreted as completion according to the study’s operational and programmatic definition rather than as full adherence to the WHO-recommended ANC8 + schedule. Finally, the binary classification of complete versus incomplete care may obscure differences between women who partially completed the continuum and those who dropped out at earlier stages. To partly address this limitation, stage-wise descriptive indicators and regression models were included to support interpretation of continuation across ANC4 + , skilled birth attendance, and early postnatal care. Future studies may consider ordered, multinomial, or other models to examine dropout patterns more explicitly.

## Conclusions

Completion of the maternal continuum of care among rural women in East Shoa Zone remained low. Nearly four-fifths of women did not complete the maternal continuum of care. The progressive decline from antenatal care attendance to skilled delivery and timely postnatal care indicates significant loss to follow-up at successive stages of the maternal care pathway. Completion of the continuum was influenced by both individual and health-system factors. Maternal education, early initiation of antenatal care, adequate antenatal care content, counseling on pregnancy danger signs, planned pregnancy, shorter travel time to a health facility, and higher facility readiness were positively associated with completion. The substantial variation observed between kebeles further indicates that community-level and health-system conditions are important determinants of continuity of care. Therefore, maternal health programs should combine demand-side strategies that improve women’s awareness, preparedness, decision-making capacity, and early engagement with services with supply-side measures that strengthen health facility readiness. In addition, qualitative research is recommended to further explore barriers to the maternity continuum of care.

## Abbreviations

ANC: Antenatal Care
AOR: Adjusted Odds Ratio
CI: Confidence Interval
CoC: Continuum of Care
EDHS: Ethiopian Demographic and Health Survey
HEWs: Health Extension Workers
LMIC: Low and Middle-Income Countries
MMR: Maternal Mortality Ratio
MNCH: Maternal, Neonatal and Child Health
MOH: Ministry of Health
OR: Odds Ratio
PNC: Postnatal Care
SBA: Skilled Birth Attendants
SDG: Sustainable Development Goal
SSA: Sub-Saharan Africa
WHO: World Health Organization
